# COVID-19-associated mortality in individuals with serious mental disorders in Sweden during the first two years of the pandemic– a population-based register study

**DOI:** 10.1186/s12888-024-05629-y

**Published:** 2024-03-07

**Authors:** Anna Gibbs, Martin Maripuu, Louise Öhlund, Micael Widerström, Niklas Nilsson, Ursula Werneke

**Affiliations:** 1 Department of Clinical Sciences, Division of Psychiatry, Sunderby Research Unit, Umeå University, Umeå, Sweden; 2 Department of Clinical Sciences, Division of Psychiatry, Umeå University, Umeå, Sweden; 3 Department of Clinical Microbiology, Umeå University, Umeå, Sweden; 4 Department of Psychiatry, Sunderby Hospital, Luleå, 97180 Sweden

**Keywords:** Coronavirus, COVID-19, Psychotic disorder, Psychosis, Bipolar disorder, Depressive disorder, Mental disorder, Mortality, Death, Risk factor

## Abstract

**Background:**

Reports at the beginning of the COVID-19 pandemic suggested differences in COVID-19-associated mortality between individuals with serious mental disorders (SMD) and the population at large.

**Aim:**

To compare the pattern of COVID-19-associated mortality in individuals with and without SMD in Sweden over the two main pandemic years.

**Methods:**

We compared the pattern of COVID-19-associated mortality in individuals with and without SMD in Sweden during 2020 and 2021. For SMD, we included psychotic disorder, bipolar disorder, and severe depression. The analysis was based on summary data from the Swedish Board of Health and Welfare covering the entire adult Swedish population.

**Results:**

The overall relative risk (RR) for experiencing a COVID-19-associated death was 1.66 (CI 1.50–1.83; *p* < 0.001) for individuals with SMD versus individuals without SMD. The corresponding RRs were 3.25 (CI 2.84–3.71; *p* < 0.001) for individuals with psychotic disorder, 1.06 (CI 0.88–1.26; *p* = 0.54) for individuals with bipolar disorder, and 1.03 (CI 0.80–1.32; *p* = 0.80) for individuals with severe depression. Compared to their respective counterparts in the non-SMD group, in the psychotic disorder and severe depression group, the RR were higher in women than in men. In the bipolar disorder group, the RR was higher in men than in women. The RR of COVID-19-associated death was generally higher in younger individuals with SMD. Individuals with psychosis between 18 and 59 years had the highest RR of COVID-19-associated death with 7.25 (CI 4.54–11.59; p<0.001).

**Conclusions:**

Individuals with SMD, and particularly those with psychotic disorders, had a higher risk of COVID-19-associated death than the general population. As this is a pattern also seen with other infections, people with SMD may be similarly vulnerable in future pandemics.

**Supplementary Information:**

The online version contains supplementary material available at 10.1186/s12888-024-05629-y.

## Background

Early into the COVID-19 pandemic, reports emerged that individuals with serious mental disorders (SMD) might have a higher risk of COVID-19-associated death than individuals without SMD [1, 2]. Since then, numerous studies have been published. Most of these have been summarised in eight meta-analyses [3–10]. Although these meta-analyses vary in risk estimates depending on SMD type, there is a consensus that individuals with SMD should be considered a high-risk group [11, 12].

When vaccines became available, several countries included individuals with SMD in the prioritised groups to benefit from special awareness and early vaccination [13]. Once prioritised, the question arose whether the mortality gap between individuals with and without SMD could narrow. However, evaluating this would require a sufficiently long-term perspective. There is still a lack of comprehensive data on COVID-19-associated mortality in individuals with SMD over the first two pandemic years. Only few studies have been published taking the required long-term perspective to cover the start of vaccination [14–20].

At present, it also remains unclear why individuals with SMD experience higher COVID-19-associated mortality. Individuals with SMD in general may experience increased somatic vulnerability. Type of mental disorder, lack of social support, a greater propensity to risk-taking behaviour, poor life-style choices and harmful substance use may all play a role [21]. Inequalities due to race/ethnicity, and inequalities regarding access to hospital care may further increase the mortality risk for some subgroups [22–25]. Regarding sex distribution, it has been generally accepted from the beginning of the pandemic that men may have a higher risk of COVID-19-associated death [18, 26, 27]. This also seems to hold true for individuals with SMD [1, 19, 20]. The sex difference may have declined with the roll-out of vaccination [18]. However, to our knowledge, studies have not explored the role of sex in relation to SMD type. If women with a particular SMD had a higher risk of COVID-19-associated death or proportionately less benefit of vaccination, this would largely go unnoticed.

Equally, it remains unclear how individuals with SMD in Sweden have fared during the COVID-19 pandemic. In Sweden, strategies and health care policies during the pandemic differed largely from most other countries. Social distancing and lockdown measures were much less strictly enforced [28]. It remains unclear whether, and if so in what way, this could have affected mortality figures and altered the impact of vaccination. We could only find two studies that examined COVID-19-associated mortality in individuals with SMD in Sweden. Both studies had a too short time horizon to assess the impact of vaccination [2, 29]. Therefore, we set up the current study to examine COVID-19-associated mortality in Sweden for the two main pandemic years.

### Aim

We conducted this study to compare the pattern of COVID-19-associated mortality in individuals with and without SMD in Sweden over the first two years of the pandemic, including the time-period when the COVID-19 vaccine became available, in relation to underlying psychiatric diagnoses, sex and age. At the beginning of the study, based on the available knowledge at the time, we had assumed that COVID-19-associated mortality would (a) be higher in individuals with SMD across all diagnostic categories, (b) be disproportionately higher in younger individuals with SMD, (c) be higher in men with SMD across all diagnostic categories, and (d) decline to a larger extent after the vaccine availability in individuals with SMD than without SMD.

## Methods

### Study design

This was a retrospective nationwide register study, based on the Swedish National Patient Register and the Swedish National Death Register, both held by the Swedish Board for Health and Welfare (Socialstyrelsen). The data obtained from the respective registers were linked through the unique personal identification number. The data used for the current analysis was collated by the Swedish Board of Health and Welfare, which then made it available as summary data in anonymised form. The study was approved by the Swedish Ethical Review Authority (Etikprövningsmyndigheten) (DNR 2020–02759, DNR 2021–05175) and conducted according to the declaration of Helsinki. As the data was only provided in anonymised summary form, individuals could not be identified and informed consent could not be obtained. This was accepted by the Swedish Ethical Review authority so that the need for consent was waived. The Swedish Board of Health and Welfare only provided the data after ethical approval had been obtained. At this point, the Swedish Board of Health and Welfare withheld data considered potentially identifiable (data withheld due to confidentiality reasons). The method adhered to the Strobe checklist [30].

### Sample

We included the entire Swedish population of at least 18 years of age by 31 Dec 2019. Cases were defined as individuals with a diagnosis of SMD; all other individuals (the rest of the population) were defined as non-SMD.

### Data sources

The study used data from the Swedish National Patient Register (Patientregistret) [31] and the Swedish National Death Register (Dödsorsaksregistret) [32]. The Swedish National Patient Register is based on diagnoses according to the International Classification of Disease, 10th revision (ICD-10) [33] for both inpatient and outpatient care in specialised medicine (secondary care). The Swedish National Patient Register was founded in 1964 and covered the whole country since 1987. As of 1998, the register recorded ICD-10 diagnoses. In 2001, the register was extended not only to cover inpatient but also outpatient specialist care [34]. Diagnoses from general practitioners (primary care) are not included in this register. Cause-of-death data were retrieved from the Swedish National Death Register, which includes all Swedish residents that have died. The cause of death was established in either primary or secondary care, depending on where the death had occurred.

### Outcomes

Our primary outcome was COVID-19-associated death, registered as such by the Swedish Board for Health and Welfare. In Sweden, the first confirmed case of COVID-19 infection was reported on 31 January 2020 [35]. The first COVID-19-related death was reported on 11 March 2020 [35]. We analysed the first two years of the pandemic by half years, using 1 January 2020 until 31 December 2021 as a time frame. We analysed COVID-19-associated deaths, as a dichotomous yes/no variable. The Swedish Board for Health and Welfare bases the criteria COVID-19-associated death on the underlying cause of death recorded on the death certificates. Two codes, U07.1 or U07.2, of ICD-10 were used. U07.1 was used when COVID-19 had been confirmed by laboratory testing irrespective of severity of clinical signs or symptoms. U07.2 was used when COVID-19 was diagnosed clinically or epidemiologically, but laboratory testing was inconclusive or not available [33].

### Exposures /Selection criteria

#### Serious mental disorder

The main exposure (case) was SMD. In principle, SMD can contain a wide range of psychiatric diagnoses. Both nature and degree matter. Using SMD in analogy to the US National Institute of Mental Health (NIHMH) term “serious mental illness” (SMI), SMD can be defined as “a mental, behavioural, or emotional disorder resulting in serious functional impairment, which substantially interferes with or limits one or more major life activities” [36]. This definition has been used by major US surveys such as the 2021 National Survey on Drug Use and Health (SAMHSA). When using this SMI definition, SAMHSA excludes developmental conditions and substance use disorders (SUD) [37].

#### Rationale for focusing on psychotic disorders, bipolar disorder, and severe depression

Considering all possible psychiatric diagnoses would have been beyond the scope of our study. We also only had access to summary data that was pre-analysed by the Swedish Board for Health and Welfare (cf. Statistical methods). This, and the relatively small number of outcomes, i.e., COVID-19-associated deaths, limited our ability to account for multiple combinations of psychiatric diagnoses. Hence, we decided to focus on those conditions, which we (a) could reasonably assume to be at the forefront of clinician’s minds, and (b) had received prominent attention in the literature. These were psychotic disorders, bipolar disorder, and severe depression [1, 2, 11, 12]. We grouped these according to the algorithm outlined in panel 1.

#### Inclusion criteria

Individuals were included in the SMD group when there were at least two registered diagnoses between 1 January 2010 and 31 December 2019, according to the algorithm outlined in panel 1. In this algorithm, psychotic diagnoses took precedence over affective diagnoses, i.e., if there was one diagnosis of psychosis, an individual was placed into the psychotic disorder group. Other variables used in the analyses were sex and age, the latter categorised into three groups: 18–59, 60–79 and 80 + years. We required two diagnoses of the included conditions according to panel 1 to increase diagnostic certainty. Using the two-diagnoses requirement has been shown to yield sufficiently sensitive and specific diagnoses for use in epidemiological studies [38]. This way, we focussed on persistent psychotic conditions and ensured the exclusion of one-off psychotic or affective states recorded in the context of other disorders, such as SUD (ICD-10 F10 category) or severe anxiety or dissociative disorders (ICD-10 F40 category) [33]. We focussed on SMD that had pre-existed in the ten years prior to the COVID-19 outbreak for two reasons, (a) SMD would most likely need to have been present for a substantial period of time to accrue clinically relevant somatic harm, and (b) SMD that had gone into remission for several years should not be included.

**Panel 1:** Panel 1. Diagnostic algorithm

Diagnosis	Definition according to the ICD-10
Psychotic disorder	At least two diagnoses of F20, F22 or F25 OR At least one diagnosis of F20, F22 or F25 AND at least one diagnosis of F30, F31, F32 or F33
Bipolar disorder	At least two diagnoses of F30 or F31 OR At least one diagnosis of F30 AND at least one diagnosis of F31, F32 or F33 OR At least one diagnosis of F31 AND at least one diagnosis of F30, F32 or F33
Severe depression	At least two diagnoses of F32.2, F32.3, F33.2, F33.3

### Statistical methods

All data were linked, anonymised, and summarised by the Swedish Board of Health and Welfare (Socialstyrelsen). The provided tabulated data included information arranged according to cases and controls on the number of individuals in each diagnostic and age groups as well as sex categorisation. When stratified data resulted in outcomes for less than five individuals, the Swedish Board of Health and Welfare withheld the information for confidentiality reasons. This missing data were set to 0 in the statistical analysis.

We analysed the frequency of COVID-19-associated deaths over the entire two-year period. For this time-frame, we compared the frequency of COVID-19-associated deaths between the SMD- and non-SMD groups, for (a) the whole group and (b) for the whole group stratified by diagnosis, sex, and age. We also examined the COVID-19-associated mortality pattern over time, dividing the two-year observation period into four half-year- periods (H), 2020H1, 2020H2, 2021H1 and 2021H2. We calculated the frequency of COVID-19-associated deaths based on the population being alive at the beginning of every half year under study. For all comparisons, we calculated risk ratios (RR). In theory, covering the entire Swedish population would not require calculation of confidence intervals (CI). However, we included CI to allow for general conclusions for populations beyond Sweden. Microsoft Excel was used to analyse the data descriptively and plot the graphs. RR, CI and p-values were calculated using OpenEpi version 3.01 [39]. Two-sided p-value < 0.05 was considered of statistical significance.

## Results

### Baseline characteristics of the sample

**Fig. 1:**
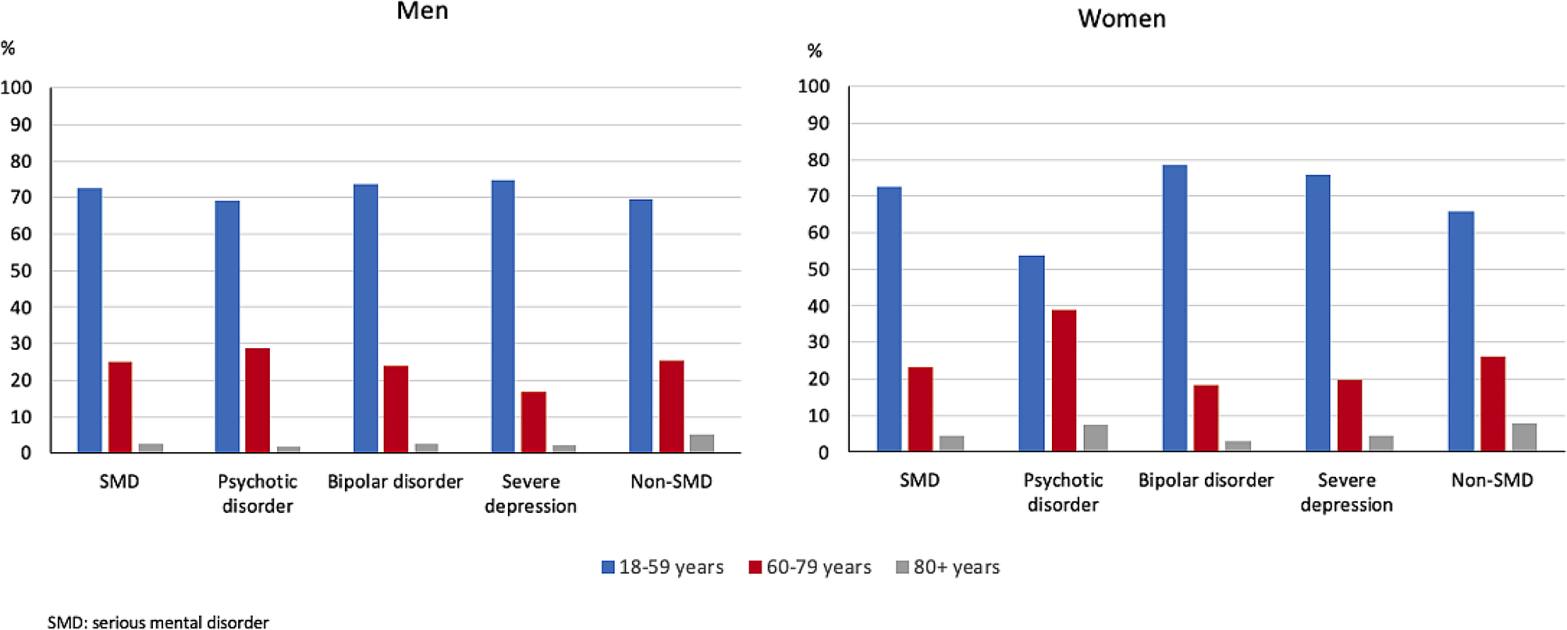
Age distribution according to diagnostic categories in men and women

The sample included in total 8,147,081 individuals, 135,973 individuals in the SMD group (1.7%) and 8,011,108 in the non-SMD group (98.3%). Further breakdown of the proportions in the SMD group was as follows: 0.5% psychotic disorder, 0.8% bipolar disorder, and 0.4% severe depression. In the SMD group, 95.5% of COVID-19 diagnoses were determined by ICD-10 code U07.1; in the non-SMD group, 96.8% of COVID-19 diagnoses were determined by ICD-10 code U07.1. The full details of the sample are presented in Table 1 (Table 1). Regarding age and sex distribution, there were about 1.5–2 times more women aged 60 to 79 years in the psychotic group than in the other groups (Fig. 1) (appendix).

**Table 1:** Demographic characteristics of the sample (*n* = 8,147,081)

	SMD (all)		Psychotic disorder		Bipolar disorder		Severe depression		non-SMD	
	*n*	%	*n*	%	*n*	%	*n*	%	*n*	%
**Total** ^**a**^										
**Total**	135,973	1.7	37,580	0.5	64,139	0.8	34,254	0.4	8,011,108	98.3
**Sex** ^**b**^										
**Men**	57,468	42.3	20,268	53.9	23,020	35.9	14,180	41.4	4,016,067	50.1
**Women**	78,505	57.7	17,312	46.1	41,119	64.1	20,074	58.6	3,995,041	49.9
**Age (years)** ^**b**^										
**18–59**	98,486	72.4	23,331	62.1	49,350	76.9	25,805	75.3	5,414,838	67.6
**60–79**	32,764	24.1	12,560	33.4	13,070	20.4	7,134	20.8	2,064,687	25.8
**80+**	4,723	3.5	1,689	4.5	1,719	2.7	1,315	3.8	531,583	6.6

^a^% calculated with total population as denominator

^b^% calculated as total number in respective diagnostic group as denominator

### COVID-19-associated deaths in SMD and non-SMD group, overall and stratified by diagnosis

In the two years under study, there were 14,704 (0.2%) COVID-19-associated deaths, 402 (0.3%) in the SMD group and 14,302 (0.2%) in the non-SMD group. The full breakdown of deaths is presented in Table 2.

**Table 2:** COVID-19-associated deaths (*n* = 14,704)

	SMD (all) *n*_deaths_ = 402		Psychotic disorder *n*_deaths_ = 218		Bipolar disorder *n*_deaths_ = 121		Severe depression *n*_deaths_ = 63		non-SMD *n*_deaths_ = 14,302	
**Sex**										
**Men**	191	47.5%	104	47.7%	59	48.8%	28	44.4%	7945	55.6%
**Women**	211	52.5%	114	52.3%	62	51.2%	35	55.6%	6357	44.4%
**Age (years)**										
**18–59**	28	7.0%	18	8.3%	5	4.1%	5	7.9%	576	4.0%
**60–79**	220	54.7%	135	61.9%	63	52.1%	22	34.9%	4212	29.5%
**80+**	154	38.3%	65	29.8%	53	43.8%	36	57.1%	9514	66.5%

The RR was 1.66 (CI 1.50–1.83; *p* < 0.001) for COVID-19-associated death in the SMD group compared to the non-SMD group. Regarding diagnoses, the RR for individuals with psychotic disorder compared to the non-SMD group was 3.25 (CI 2.84–3.71; *p* < 0.001). The respective RR for individuals with bipolar and severe depression were 1.06 (CI 0.88–1.26; *p* = 0.54) and 1.03 (CI 0.80–1.32; *p* = 0.80).

Compared to the respective non-SMD strata, both men and women with SMD had a similarly increased risk of COVID-19-associated death, with respective RR of 1.68 (CI 1.46–1.94; *p* < 0.001) and 1.69 (CI 1.47–1.94; *p* < 0.001). Regarding age, the RR between SMD and non-SMD strata were 2.67 (CI 1.83–3.91; *p* < 0.001) for individuals aged 18–59 years, 3.29 (CI 2.88–3.77; *p* < 0.001) for individuals aged 60–79 years, and 1.82 (CI 1.56–2.13; *p* < 0.001) for individuals 80 + years (appendix).

### COVID-19-associated deaths in the individual diagnostic groups and the non-SMD group, stratified by age and sex

**Fig. 2:**
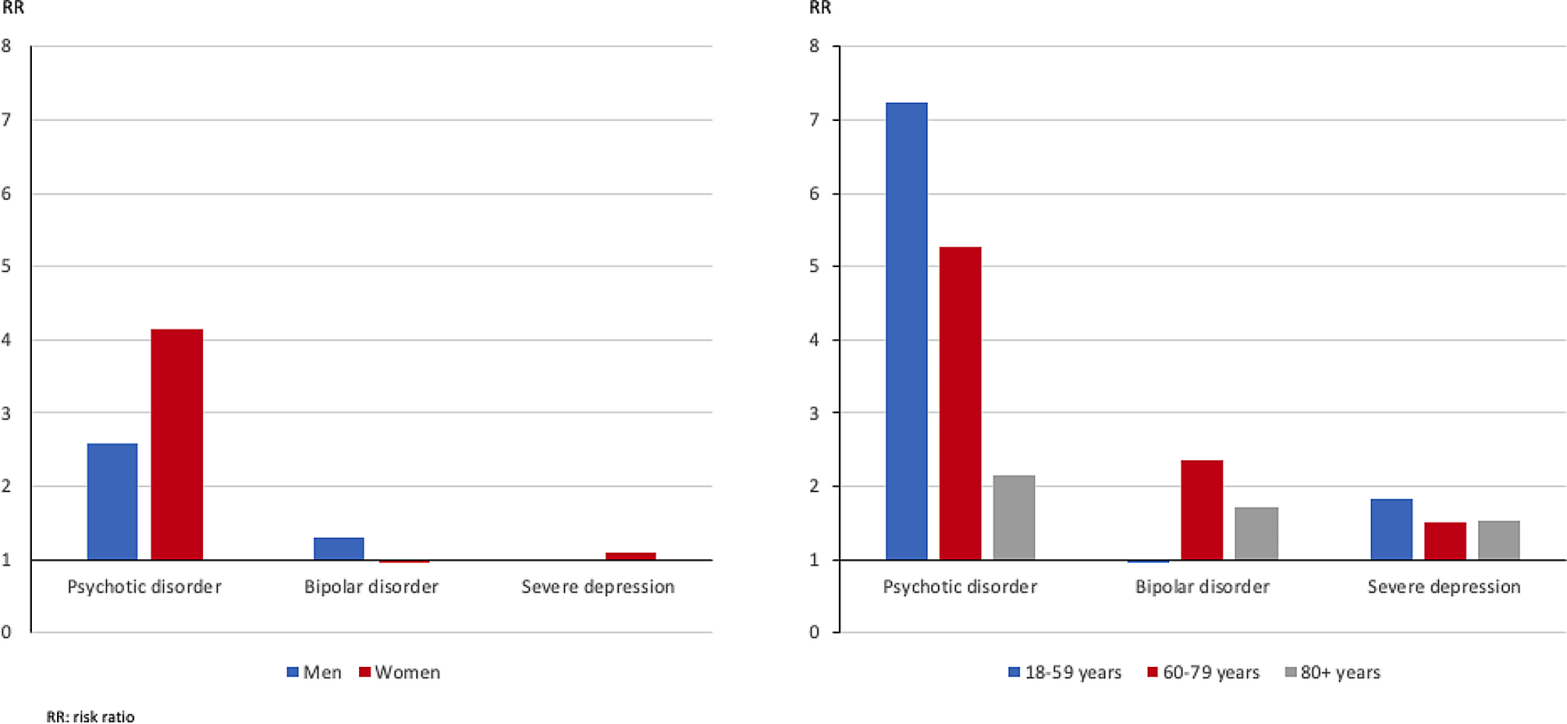
Risk ratios for COVID-19-associated deaths in the SMD compared with the non-SMD group as the baseline, stratified by sex and age

Compared to the respective non-SMD strata, in the psychotic disorder group, the RR was 4.14 (CI 3.44–4.98; *p* < 0.001) for women and 2.59 (CI 2.14–3.16; *p* < 0.001) for men. For the other diagnostic groups, the RR were close to one. Regarding age, compared to the respective non-SMD strata, the RR pattern was mixed. In the psychotic disorder group, the RR was highest in the age groups of 18- to 59-year-olds with 7.25 (CI 4.54–11.59; *p* < 0.001), followed by 60 to 79 years-olds with 5.27 (CI 4.44–6.25; *p* < 0.001), and the 80 + year-olds with 2.15 (CI 1.69–2.73; *p* < 0.001). In the bipolar disorder group, the respective RR were 2.36 (CI 1.84–3.01; *p* < 0.001) in 60- to 79-year-olds, 1.72 (CI 1.32–2.25; *p* < 0.001) in the 80 + years-olds, and 0.95 (CI 0.40–2.30; *p* = 0.97) in the 18 to 59 years-olds. In the severe depression group, the respective RR were 1.82 (CI 0.76–4.39; *p* = 0.21) in the 18 to 59 years-olds, 1.53 (CI 1.12–2.11; *p* = 0.02) in the 80 + year-olds, and 1.51 (CI 0.99–2.30; *p* = 0.07) in the of 60 to 79 year (Fig. 2) (appendix).

### COVID-19-associated mortality pattern over time

**Fig. 3:**
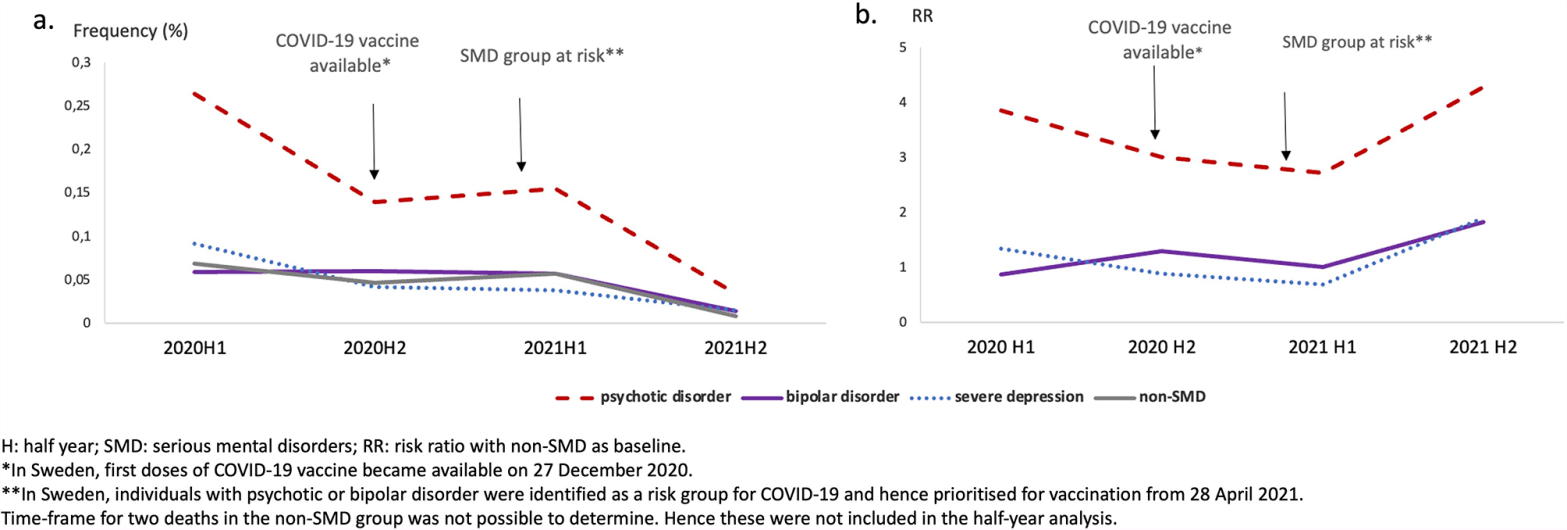
Frequency (**a**) and risk ratios (**a**) of COVID-19-associated deaths, between 1st January 2020 and 31st December 2021 in the serious mental disorder and non-serious mental disorder groups

In all diagnostic groups, the highest proportion of COVID-19-associated deaths was seen during the first half year of 2020. Compared to the non-SMD group, the RR for all individuals with psychotic disorder was consistently increased three to four times. For all three psychiatric diagnoses, the RR was highest in the second half year of 2021, with a RR of 4.27 (CI 2.41–7.56; *p* < 0.001) in individuals with psychotic disorder, 1.82 (CI 0.94–3.51; *p* = 0.10) in individuals with bipolar disorder, and 1.89 (CI 0.78–4.54; *p* = 0.18) in individuals with severe depression. In the final half-year of 2021 (2021H2), the mortality had sunk to less than 0.1% for all diagnostic groups (Fig. 3) (appendix).

## Discussion

Our study showed that over the first two years of the pandemic, the proportion of COVID-19-associated deaths was higher in individuals with SMD than in individuals without SMD. Although individuals with SMD had higher COVID-19-associated mortality in relative terms, the actual numbers of deaths were low. Our results were mainly driven by a higher proportion of deaths in individuals with psychotic disorder. Regarding age, younger individuals with psychotic or bipolar disorder were disproportionally affected. In the psychotic disorder group, the RR of COVID-19-associated death was highest in the age groups of 18 to 59 and 60- to 79-year-olds. In the bipolar disorder group, the RR of COVID-19-associated death was highest in the age groups of 60- to 79-year-olds. Contrary to what we had expected, in the psychotic disorder group, the RR of COVID-19-associated death for women with SMD versus women without SMD was higher than the corresponding RR for men with SMD versus men without SMD. Although absolute numbers of COVID-19-associated death declined dramatically after the introduction of the vaccination programme, the RR remained higher for all three diagnostic SMD groups. The RR increased again in the second half of 2021.

### Comparison with other studies

Overall, our findings over this two-year period are in line with the reports from the very beginning of the pandemic [1, 2]. The findings are also in line with subsequent reports, which now have been summarised in eight meta-analyses (panel 2) [3–10]. These meta-analyses give risk estimates of RR or odds ratios (OR), ranging from 1.38 to 2.0 for any mental disorder [7–10] and 1.67 for SMD [7] (panel 2). This is in line with our RR for SMD in total of 1.66. Four of these meta-analyses addressed psychotic disorders/ schizophrenia. In these, the OR ranged from 2.05 to 2.28 [3, 5, 8, 10]. This is lower than our RR for psychotic disorders of 3.25. Three meta-analyses addressed mood disorders. In these, the OR ranged from 1.50 to 1.99 [5, 6, 10]. In contrast to these findings, in our study, the RR for bipolar disorder and severe depression only marginally, and not significantly, increased. Our diagnostic algorithm with the requirement of two SMD diagnoses may have biased our sample towards the severe end of the SMD spectrum. This may have contributed to higher RR for the psychotic disorder groups. Our diagnostic algorithm was also set towards psychotic disorders taking precedence over affective disorders. This possibly reduced the risk estimates for the bipolar disorder and severe depression group, which may have eroded any significant differences to the reference group. Notably, the proportion of severe depression was relatively low. This may again have been due to our algorithm; two severe episodes of depression were required to qualify. Individuals with only one severe episode of depression would have been allocated to the bipolar or psychotic disorder group if there was a relevant second diagnosis. Otherwise, they would have been excluded.

**Panel 2:** Risk estimates of COVID-19-associated mortality in individuals with mental disorders from eight meta-analyses against baseline without mental disorder

Study	Studies included until	Outcome	*N* studies	Result OR/RR (95% CI)^a^
*Any mental disorder*				
Fond et al., 2021 [7]	12 February 2021	Death, OR	21	**1.38 (1.15–1.65), adjusted**
Liu et al., 2021 [8]	7 July 2021	Death, OR	28	**1.47 (1.26–1.72)**
Toubasi et al., 2021 [9]	15 February 2021	Death + severity, OR	5	**1.52 (1.20–1.93), fully adjusted**
Vai et al., 2021 [10]	5 March 2021	Death, OR	21	**2.00 (1.58–2.54)**
*Serious (severe) mental disorder*				
Fond et al., 2021 [7]	12 February 2021	Death, OR	5	**1.67 (1.02–2.73), adjusted**
*Psychosis/ psychosis spectrum disorder*				
Molero et al., 2023 [5]	27 June 2023	Death, OR	13	**2.15 (1.68–2.75)**
Vai et al., 2021 [10]	5 March 2021	Death, OR	4	**2.05 (1.37–3.06)**
*Schizophrenia*				
Pardamean et al., 2022 [3]	15 November 2021	Death, RR	10	**2.22 (1.54–3.20)**
Liu et al., 2021 [8]	7 July 2021	Death, OR	8	**2.28 (1.40–3.73)**
*Mood disorder: depression and/or bipolar disorder*				
Molero et al., 2023 [5]	27 June 2023	Death, OR	14	**1.50 (1.31–1.71)**
Ceban et al., 2021 [6]	1 February 2021	Death, OR	12	**1.51 (1.34–1.69)**
Vai et al., 2021 [10]	5 March 2021	Death, OR	6	**1.99 (1.46–2.71)**
*Neurodevelopmental disorder*				
Molero et al., 2023 [5]	27 June 2023	Death, OR	2	1.26 (0.77–2.05)
*Anxiety disorder*				
Molero et al., 2023 [5]	27 June 2023	Death, OR	6	1.14 (0.72–1.80)
Liu et al., 2021 [8]	7 July 2021	Death, OR	4	1.16 (0.75–1.79)
Vai et al., 2021 [10]	5 March 2021	Death, OR	3	1.07 (0.73–1.56)
*Substance use disorder*				
Molero et al., 2023 [5]	27 June 2023	Death, OR	11	**1.45 (1.12–1.87)**
Vai et al., 2021 [10]	5 March 2021	Death, OR	4	**1.76 (1.27–2.44)**
*Opioid use disorder*				
Behnoush et al. 2022 [4]	December 2021	Death, OR	2	**1.52 (1.27–1.82)**

^a^Statistically significant results in bold type

A recently published study from the US, not included in the meta-analyses, compared overall mortality in 5,140,619 older adults before and during the pandemic. 246,422 deaths occurred in 2020, which was a 14.5% increase over expected. The largest increases were observed in individuals with a diagnosis of schizophrenia with a 32.4% increase of deaths and in individuals with a diagnosis of bipolar disorder with a 25.4% increase. There were also excess deaths in individuals with depression and anxiety, albeit to a lesser extent. Excess mortality was 17.0% in individuals with depression and 15.4% in individuals with anxiety. The same study found that psychiatric diagnoses were associated with higher COVID-19 infection rates. Therefore, at least partly, the excess deaths were considered linked to COVID-19 [15]. Another US study based on electronic health records from 116,498 individuals with COVID-19 events between March 2020 and February 2021 found that pre-existing psychosis/ bipolar disorder increased the risk of COVID-19-associated death 1.4- fold. Depression, anxiety, and ADHD made no significant difference [40].

For Sweden, we could only find two studies that examined COVID-19-associated mortality in individuals with SMD. Both studies had a relatively short time horizon, hence, neither could assess vaccination impact. The first study, originating from our own research group, explored COVID-19-associated mortality in individuals with SMD for the first three months of the pandemic (from 11 March 2020 to 15 June 2020). SMD group included individuals with pre-existing psychotic or bipolar disorder. Compared to the non-SMD group the odds were two-fold. For the age-group between 60 and 79 years, the odds were four-fold. This study did not distinguish between various types of SMD, neither explored potential sex differences [2]. The second study explored COVID-19-associated mortality for a 10,5-month period (from 1 March 2020 to 14 January 2021). The endpoint of this study only covered the first three weeks of the Swedish vaccination campaign, at which point only few individuals had received the vaccine. This study was based on primary and secondary care data and addressed common mental disorders (CMD), SUD, and SMD. CMD concerned depression and/or anxiety or stress related disorders. SUD concerned alcohol and/or other substances. SMD was defined as “non-affective and affective psychotic disorders including bipolar disorder with (SMD+) and without (SMD) possible comorbidities with other mental disorders” [29]. In this study, the fully adjusted hazard ratio (HR) for COVID-19-associated death was 1.2 for CMD, 1.3 for SUD, 1.5 for SMD, and 2.9 for SMD+ [29].

In contrast to most other countries, Sweden did not employ complete lockdown measures. Ultimately, this may not have led to higher mortality. One study ranked 14 European countries in regard to excess all-cause mortality between 2020 and 2022 by stringency of lockdown. Rank 1 indicated the highest excess mortality and rank 14 the lowest. According to this study, Sweden had employed the least stringent lockdown but ranked relatively low on excess all-cause mortality at both endpoints. In the first year of the pandemic (2020), Sweden ranked 9/14 with 85 excess deaths/ 100,000 population. By the end of 2022, Sweden ranked 12/14 with 158 excess deaths/ 100,000 population [28]. However, it remains unclear how the different Swedish lockdown policies may have impacted on individuals with SMD in terms of access to mental health and somatic care. One study from Southern Sweden explored access to mental health care at the beginning of the pandemic, using changes in the dispensed amount of common psychotropic medications as a proxy. This study concluded that access to mental health care may not have been impaired [41]. Another study examined the accessibility of intensive care beds in terms of availability and geographic distance in 14 countries at the beginning of the pandemic. This study found Sweden to have the lowest accessibility to intensive care beds [42]. However, we could not find any study exploring inequities in access to intensive care in Sweden. Two studies from France and Spain suggest that individuals with SMD had less access to salvage therapy and critical care [23, 24].

### Vulnerability of individuals with serious mental disorders

One question of interest is whether the higher mortality risk can be interpreted as a vulnerability of individuals with SMD to COVID-19 infection in particular, or as confirmation of a shorter life expectancy and higher vulnerability to somatic disorders in general. Race/ethnicity may also play a role. Although this question was not the aim of our study it could be noted that the higher mortality risk associated with COVID-19 was in line with the mortality risk seen with several other somatic conditions [43, 44]. In our previous work, we have shown similarly increased odds for death and hospitalisation related to influenza, pneumonia, or sepsis for individuals with SMD [45]. Most likely, individuals with SMD in general and psychotic disorders in particular have other, non-specific, somatic risk factors that put them at higher risk. The examination of these was beyond the scope of our study. Such general risk factors may include comorbid conditions such as cardiovascular diseases, diabetes, and obesity [21, 46], lifestyle factors such as smoking [47], or other substance use [21, 48], or psychotropic agents such as antipsychotics or benzodiazepines [46, 49]. The meta-analysis by *Vai et al.* showed that antipsychotics were significantly associated with a higher risk of COVID-19-associated death (adjusted OR 2.43, CI 1.81–3.25). So were anxiolytics (adjusted OR 1.47, 1.15–1.88) [10]. Antipsychotics may increase the risk of cardiovascular events and thromboembolism [50]. Anxiolytics, particularly, benzodiazepines may increase respiratory risks [50]. Differences in immunological profile [51], barriers to somatic care [52], socioeconomic [53], social and environmental factors may all play a role. However, discontinuing withholding psychotropic medications patients need, for fear of COVID-19-associated deaths, may do more harm than good. The increases in risk of relapse and suicide may outweigh any potential gains in physical health [21, 54].

It has also been hypothesised that some psychotropic drugs could also be protective. A recent meta-analysis examined such “psychotropic drug repurposing” for COVID-19 [55]. This meta-analysis found an increased risk of COVID-19-associated death with antipsychotics. There was no change with antidepressants. Based on two studies, the antidepressant fluvoxamine was associated with a significant reduction of COVID-19-associated mortality (OR 0.15, CI 0.02–0.95) [55]. A further study, not included in this meta-analysis but aforementioned in our discussion, found a reduced risk of COVID-19-associated death with antidepressants (OR 0.70, CI 0.51–0.96) [40]. However, the most recent meta-analysis evaluating the effectiveness of fluvoxamine for COVID-19 outpatient management did not find any significant reduction of COVID-19 associated mortality (RR 0.73; CI 0.42–1.28) [56]. Even lithium has been suggested as a potentially protective agent based on purported antiviral properties [57] and in one observational study based on serum lithium concentrations [58]. At present, psychotropic drug-repurposing for COVID-19 remains controversial with conflicting results and lack of demonstrated mechanisms of action.

### Mortality pattern over time

Even if there were differences in COVID-19-associated mortality between individuals with SMD and the population at large, it remains unclear whether this pattern has persisted over time. To address this, we explored mortality figures for the two pandemic years, over the four distinct half-year periods. The absolute number of deaths was highest in the first half-year of the pandemic (2020H1) across all diagnostic groups and dropped substantially during the first half-year of 2021 (2021H1), which corresponded with vaccine prioritisation time for individuals with SMD. In Sweden, individuals with schizophrenia and bipolar disorder were prioritised for vaccination from 28 April 2021 [59]. Interestingly, the RR for all diagnostic groups remained elevated over time. It was highest at the end of our study period (2021H2), at which point, the absolute number of deaths had dropped significantly. This suggests that the vaccine took effect in all four groups, but individuals with SMD did not derive a proportionally larger benefit from the vaccination than non-SMD.

Our findings are in line with a study from Israel. This study examined COVID-19 mortality before and after vaccination in about 25,539 individuals with schizophrenia and 25,539 controls up till 30 April 2021. The HR was 2.52 for individuals with schizophrenia and survival declined more steeply as the study progressed. Although, mortality rates substantially declined in both study groups after introduction of vaccination [16]. Another recent study suggests that groups prioritised for vaccination, including individuals with SMD, experienced larger decreases in COVID-19-associated mortality than the population in general [18]. In two other studies, however, COVID-19-associated mortality seems to have declined in only comparable rates in individuals with and without SMD [15, 19, 20]. For Sweden, we have not found any other studies assessing the impact of vaccination.

We do not know how good vaccination uptake became among individuals with SMD in our region. Studies from Israel [16, 17] and England [60] show lower vaccination coverage among people with SMD. However, vaccination programmes can unfold their benefits in two ways, both as direct individual protection, and indirect herd protection. Whereas individual protection is nearly immediate, herd protection may be delayed. Our observation period may have been too short to capture a herd effect. Individuals with SMD are also more likely to suffer from breakthrough COVID-19 infections [61, 62], which could possibly reflect in higher RR of COVID-19-associated mortality observed at the end of 2021. Additional studies are needed to address whether individuals with SMD may be more vulnerable to newly emerging variants because of their adverse physical risk profile.

### Sex differences

There were differences in mortality between men and women. Particularly women with psychotic disorders had a higher RR than men when compared with their respective counterparts in the non SMD group. There were more women in the SMD group. However, comparing men and women in the SMD group based on ratios with their respective counterparts, i.e., men and women in the non-SMD group, implies that the sex ratio should not have influenced the results. Besides, there were proportionally fewer women than men in the psychotic disorder group. The reason for this particularly higher mortality risk in women with psychotic disorder remains unclear. One possibility is that women experience more adverse effects with an adverse metabolic profile when taking antipsychotics, particularly when taking olanzapine or clozapine [63]. They may also have a higher risk of thromboembolism when taking oral contraceptives. Another possibility was age as a confounding factor; women with psychosis tended to be older. Specifically, there were more women with psychosis aged 60 − 79 and 80 + years than in the other diagnostic groups.

### Age differences

There were also differences in mortality between the age groups. The risk of COVID-19-associated death was consistently increased in all age groups with mostly higher RR in the younger age groups. Possibly, individuals with SMD, and particularly individuals with psychotic disorders have a higher burden of somatic comorbidities and experience such at a younger age. A similar shift in the 60–79 years population was previously observed by us [2] and in the meta-analysis by Liu et al. [8]. A particularly high physical health multimorbidity in individuals with psychotic disorder, aged 18 to 44 years has also previously been observed [64]. Therefore, younger individuals with SMD with COVID-19 infection may require closer medical attention than their younger age would suggest. However, even in older individuals, SMD may persist as a risk factor for COVID-19-associated death.

### Strengths and limitations

The major strength of our study was its large sample size covering the entire Swedish population aged 18 years and older. As our sample covered the entire Swedish population aged 18 and older rather than a random sample of the Swedish population, our data were statistically certain; all observations to be made were included. Study groups were carefully selected according to diagnosis, with at least two registered diagnoses between 2010 and 2019.

However, our study has several limitations. The data were provided as retrospective summary tables from the Swedish Board of Health and Welfare. Therefore, it was not possible to adjust for deaths as they occurred. This would have required data on individual level. Access to data on individual level would also have permitted adjustment for sex, age, severity of illness, vaccination status, psychiatric and somatic comorbidities, and use of psychotropic medications. But given the size, of the population, there were only few deaths. Most likely, further adjustments would not substantially have changed the results. At the same time, given the small number of outcomes, adjusting for a large number of variables would have resulted in overfitting. Our diagnostic algorithm with the requirement of two diagnoses of the diagnostic categories outlined in panel 1, may have biased our sample toward the severe end of the SMD spectrum and more towards psychotic disorders. This may have led to an overestimate of RR in the psychotic disorder group and to an underestimate of the RR in the affective disorder groups. Our findings, however, highlight an increased risk of COVID-19-associated death in individuals with SMD when there is a psychotic component present, irrespective of the final diagnosis.

## Conclusion

Our study confirms that individuals with SMD and particularly individuals with psychosis are a high-risk group for COVID-19-associated death. COVID-19 adverse outcomes are often associated with old age and male sex. Therefore, clinicians and public health doctors may easily lose sight of women with psychosis and middle-aged individuals with SMD despite their increased risk of COVID-19-associated death. Although COVID-19-associated mortality decreased with time, it may increase again in individuals with SMD. Targeted public health interventions, such as measures to increase vaccine uptake, need to be maintained over a long time to ensure that the mortality gap between people with SMD and the rest of the population does not increase in the future. Decreasing the mortality gap for individuals with SMD will also be a challenge in future pandemics. Our findings can inform policy makers in preparation for the next one.

### Electronic supplementary material

Below is the link to the electronic supplementary material.

Supplementary Material 1

## Data Availability

The summary tables provided by the Swedish Board of Health and Welfare are already included in this article. Requests for the original summary tables provided will be taken up with Swedish Board of Health and Welfare.

## Abbreviations

ADHD: Attention Deficit Hyperactivity Disorder
CI: Confidence interval
COVID-19: Coronavirus Disease 2019
ICD-10: International Classification of Disease, 10th revision
OR: Odds ration
RR: Relative Risk
SAMHSA: National Survey on Drug Use and Health
SMD: Serious Mental Disorders
SMI: Serious mental illness
SUD: Substance use disorder
UK: United Kingdom
US: United States
