# Supplementary material for: COVID-19-associated mortality in individuals with serious mental disorders in Sweden during the first two years of the pandemic– a population-based register study
Source: BMC Psychiatry. 2024 Mar 7;24:189. doi: 10.1186/s12888-024-05629-y (PMC10921643; doi:10.1186/s12888-024-05629-y)
Supplement: Supplementary file 1 — Supplementary Material 1 [file 12888_2024_5629_MOESM1_ESM.doc]

**Appendix**

**Data underlying figure 1: Age distribution according to diagnostic categories in men and women**

|  | **SMD (all)** | | **Psychotic disorder** | | **Bipolar disorder** | | **Severe depression** | | **non-SMD** | |
| --- | --- | --- | --- | --- | --- | --- | --- | --- | --- | --- |
| **Age, years** | ***n*** | ***%*** | ***n*** | ***%*** | ***n*** | ***%*** | ***n*** | ***%*** | ***n*** | ***%*** |
| **Men** | | | | | | | | | | |
| **18 – 59** | 41582 | 72,4 | 14018 | 69.2 | 16973 | 73,7 | 10591 | 74,7 | 2785465 | 69,4 |
| **60 – 79** | 14509 | 25,2 | 5849 | 28.9 | 5499 | 23,9 | 3161 | 16,8 | 1016092 | 25,3 |
| **80+** | 1377 | 2,4 | 401 | 2.0 | 548 | 2,4 | 428 | 2,2 | 214510 | 5,3 |
| **Women** | | | | | | | | | | |
| **18 – 59** | 56904 | 72,5 | 9313 | 53,8 | 32377 | 78,7 | 15214 | 75,8 | 2629373 | 65,9 |
| **60 – 79** | 18255 | 23,3 | 6711 | 38,8 | 7571 | 18,4 | 3973 | 19,8 | 1048595 | 26,2 |
| **80+** | 3346 | 4,3 | 1288 | 7,4 | 1171 | 2,8 | 887 | 4,4 | 317073 | 7,9 |

**Data underlying Figure 2: Risk ratios for COVID–19–associated deaths in the SMD compared with the non–SMD group as the baseline, stratified by sex and age**

|  | **SMD**  **(all diagnoses)**  **n = 135,973** | | | **Psychotic disorder**  **n = 37,580** | | | **Bipolar disorder**  **n = 64,139** | | | **Severe depression**  **n = 34,254** | | | **non-SMD**  **n = 8,011,108** | |
| --- | --- | --- | --- | --- | --- | --- | --- | --- | --- | --- | --- | --- | --- | --- |
| **Total^a^** | | | | | | | | | | | | | | |
|  | ***n*** | ***%*** | ***RR^c^*** | ***n*** | ***%*** | ***RR^c^*** | ***n*** | ***%*** | ***RR^c^*** | ***n*** | ***%*** | ***RR^c^*** | ***n*** | ***%*** |
| **Total** | 402 | 0.3 | 1.66  (CI 1.50–1.83)  p<0.001 | 218 | 0.6 | 3.25  (CI 2.84–3.71)  p<0.001 | 121 | 0.2 | 1.06  (CI 0.88–1.26)  p=0.54 | 63 | 0.2 | 1.03  (CI 0.80–1.32)  p=0.80 | 14302 | 0.2 |
| **Sex^b^** | | | | | | | | | | | | | | |
| **Men** | 191 | 47.5 | 1.68  (CI 1.46–1.94)  p<0.001 | 104 | 47.7 | 2.59  (CI 2.14–3.16) p<0.001 | 59 | 48.8 | 1.30  (CI 1.0–1.67) p=0.05 | 28 | 44.4 | 1.00  (CI 0.69–1.45) p=0.98 | 7945 | 55.6 |
| **Women** | 211 | 52.5 | 1.69  (CI 1.47–1.94)  p<0.001 | 114 | 52.3 | 4.14  (CI 3.44–4.98) p<0.00) | 62 | 51.2 | 0.95  (CI 0.74–1.22)  p=0.69 | 35 | 55.6 | 1.10  (CI 0.79–1.53)  p=0.58 | 6357 | 44.4 |
| **Age (years)^b^** | | | | | | | | | | | | | | |
| **18 – 59** | 28 | 7.0 | 2.67  (CI 1.83–3.91)  p<0.001 | 18 | 8.3 | 7.25  (CI 4.54–11.59)  p<0.001 | 5 | 4.1 | 0.95  (CI 0.40–2.30)  p=0.97 | 5 | 7.9 | 1.82  (CI 0.76–4.39)  p=0.21 | 576 | 4.0 |
| **60 – 79** | 220 | 54.7 | 3.29  (CI 2.88–3.77)  p<0.001 | 135 | 61.9 | 5.27  (CI 4.44–6.25)  p<0.001 | 63 | 52.1 | 2.36  (CI 1.84–3.01)  p<0.001 | 22 | 34.9 | 1.51  (CI 0.99–2.30)  p=0.07 | 4212 | 29.5 |
| **80+** | 154 | 38.3 | 1.82  (CI 1.56–2.13)  p<0.001 | 65 | 29.8 | 2.15  (CI 1.69–2.73)  p<0.001 | 53 | 43.8 | 1.72  (CI 1.32–2.25)  p<0.001 | 36 | 57.1 | 1.53  (CI 1.12–2.11)  p=0.02 | 9514 | 66.5 |
| ^a^% calculated with total in group as denominator  ^b^%calculated as total number in respective diagnostic group as denominator  ^c^RR calculated with controls as baseline | | | | | | | | | | | | | | |

**Data underlying figure 3:** **Frequency and risk ratios of COVID–19–associated deaths, between 1^st^ January 2020 and 31^st^ December 2021 in the severe mental disorders and non–SMD groups**

| **Half–year period** | **SMD**  **N=402** | | | **Psychotic disorder**  **N=218** | | | **Bipolar Disorder**  **N=121** | | | **Severe depression**  **N=63** | | | **non-SMD**  **N=14300^a^** | |
| --- | --- | --- | --- | --- | --- | --- | --- | --- | --- | --- | --- | --- | --- | --- |
|  | ***n*** | ***%*** | ***RR*** | ***n*** | ***%*** | ***RR*** | ***n*** | ***%*** | ***RR*** | ***n*** | ***%*** | ***RR*** | ***n*** | ***%*** |
| **2020 H1** | 168 | 0.12 | RR=1.81  (CI 1.55–2.11)  p<0.001 | 99 | 0.26 | RR=3.86  (CI 3.16–4.70)  p<0.001 | 38 | 0.06 | RR=0.87  (CI 0.63–1.19)  p=0.39 | 31 | 0.09 | RR=1.33  (CI 0.93–1.89) p=0.13 | 5472 | 0.07 |
| **2020 H2** | 103 | 0.08 | RR=1.65  (CI 1.36–2.01)  p<0.001 | 51 | 0.14 | RR=3.0  (CI 2.27–3.94)  p<0.001 | 38 | 0.06 | RR=1.29  (CI 0.93–1.77)  p=0.13 | 14 | 0.04 | RR=0.88  (CI 0.52–1.5)  p=0.67 | 3692 | 0.05 |
| **2021 H1** | 105 | 0.08 | RR=1.38  (CI 1.14–1.67)  p=0.002 | 56 | 0.15 | RR=2.71  (CI 2.08–3.52)  p<0.001 | 36 | 0.06 | RR=1.00  (CI 0.72–1.38)  P=0.99 | 13 | 0.04 | RR=0.68  (CI 0.39–1.16) p=0.14 | 4516 | 0.06 |
| **2021 H2** | 26 | 0.02 | RR=2.40  (CI 1.61–3.58)  p<0.001 | 12 | 0.03 | RR=4.27  (CI 2.41–7.56)  p<0.001 | 9 | 0.01 | RR=1.82  (CI 0.94–3.51)  p=0.10 | 5 | 0.02 | RR=1.89  (CI 0.78–4.54), p=0.18 | 620 | 0.01 |

^a^Time-frame for two deaths in the non-SMD group was not possible to determine. Hence these were not included in the half-year analysis.
